# Recurrent Episodes of New-Onset Supraventricular Tachycardia in Severe Early-Onset Pre-eclampsia and Fetal Growth Restriction: A Report of a Complex Cardio-Obstetric Case

**DOI:** 10.7759/cureus.113984

**Published:** 2026-08-05

**Authors:** Aiswarya Kaladharan Nair, Rupak Kumar Sarkar, Ajesh Sankar

**Affiliations:** 1 Department of Obstetrics and Gynaecology, Barnsley Hospital NHS Foundation Trust, Barnsley, GBR

**Keywords:** cardio-obstetrics, fetal growth restriction, high-risk pregnancy, maternal medicine, multi-disciplinary teams, pre-eclampsia, supraventricular tachycardia (svt)

## Abstract

Supraventricular tachycardia (SVT) is one of the commonly reported sustained arrhythmias in pregnancy. The rapid progression from early-onset fetal growth restriction (FGR) to severe pre-eclampsia is a well-recognised manifestation of placental dysfunction. The coexistence of both leads to significant maternal and fetal morbidity, adding substantial complexity to management.

We present the case of a 27-year-old primigravida of Caucasian origin with an otherwise uncomplicated pregnancy who attended at 28 weeks' gestation solely because of a maternal concern that her baby appeared small. This seemingly routine presentation initiated a rapid sequence of events, culminating in the diagnosis of severe early-onset FGR, fulminant pre-eclampsia and recurrent new-onset SVT.

Her clinical course was characterised by simultaneous maternal and fetal deterioration, creating significant management challenges in the context of severe hypertension, placental dysfunction and fetal compromise. Following delivery by caesarean section for worsening maternal and fetal status, she required intensive care admission, repeated administration of adenosine and ongoing multidisciplinary management to achieve cardiovascular stabilisation. Recurrent episodes of SVT persisted into the postpartum period, necessitating further specialist input and escalation to the regional Maternal Medicine Network. She was subsequently stabilised on bisoprolol and enalapril, with no further documented SVT episodes before discharge.

This case highlights the rare coexistence of severe placental disease and recurrent episodes of new-onset SVT in pregnancy, and the complexities associated with balancing maternal cardiovascular stability against fetal well-being. It underscores the importance of early multidisciplinary involvement, including obstetric, cardiology, anaesthetic, critical care and maternal medicine teams. Furthermore, it demonstrates that delivery is not always the definitive solution to complex obstetric pathology, as significant maternal cardiovascular morbidity may persist beyond the immediate postpartum period. Finally, the case emphasises the importance of listening to maternal concerns regarding fetal well-being, even when initial assessments are reassuring, and recognising the need for ongoing psychological support and counselling following traumatic pregnancy complications.

## Introduction

Supraventricular tachycardia (SVT) is one of the most common sustained arrhythmias encountered during pregnancy [1]. In a large cohort study, paroxysmal SVT occurred in approximately 24 per 100,000 pregnancies [2]. Arrhythmias that result in hospitalisation are even less common and therefore not well documented [2]. The aetiology of SVT is multifactorial and includes physiological changes of pregnancy, including hemodynamic and autonomic changes that predispose women to it [3]. Many pregnant patients presenting with SVT have no previous congenital or structural heart disease [3]. Assessment and management of maternal arrhythmias require consideration of both maternal haemodynamic status and fetal wellbeing, with multidisciplinary cardio-obstetric input recommended in complex cases [4].

Early-onset fetal growth restriction (FGR) is strongly associated with placental insufficiency and hypertensive disorders of pregnancy and may precede the clinical manifestations of pre-eclampsia [5,6]. Diagnosis and management of these conditions require careful assessment of fetal growth, Doppler abnormalities and maternal condition to optimise the timing of delivery [6]. The coexistence of severe placental disease, uncontrolled hypertension and maternal cardiac arrhythmia presents significant management challenges and necessitates coordinated multidisciplinary care. Although both severe placental disease and SVT are recognised complications of pregnancy, reports describing their coexistence are limited, and whether this represents a causal relationship or a coincidental association remains uncertain. 

We present the case of a 27-year-old woman of Caucasian origin with an otherwise uncomplicated pregnancy who attended at 28 weeks' gestation driven solely by a maternal concern that her baby appeared small. This seemingly routine presentation initiated a rapid sequence of events, from the diagnosis of severe early-onset FGR to fulminant pre-eclampsia requiring intensive maternal and fetal surveillance. Her clinical course was further complicated by recurrent, newly diagnosed SVT, creating a significant management challenge in the context of severe hypertension, placental dysfunction, and fetal compromise. Following delivery by caesarean section for worsening maternal and fetal status, she required intensive care admission, repeated adenosine administration and multidisciplinary management to achieve cardiovascular stabilisation.

This case highlights the unique clinical challenge of managing the simultaneous deterioration of maternal and fetal status, necessitating close collaboration between obstetric, cardiology, anaesthetic and critical care teams both antenatally and postnatally. We present this case to illustrate the importance of multidisciplinary management in women with high-risk obstetric and medical conditions.

## Case presentation

A 27-year-old primigravida of Caucasian origin presented to the antenatal clinic at 28+3 weeks’ gestation with concerns that her baby felt small. She had been classified as low risk at booking, with no significant medical or surgical history, a normal body mass index (BMI) of 24 kg/m², and was receiving midwifery-led care. A symphysis-fundal height measurement performed at 28 weeks plotted appropriately on the customised growth chart. She was otherwise asymptomatic.

**Figure 1:**
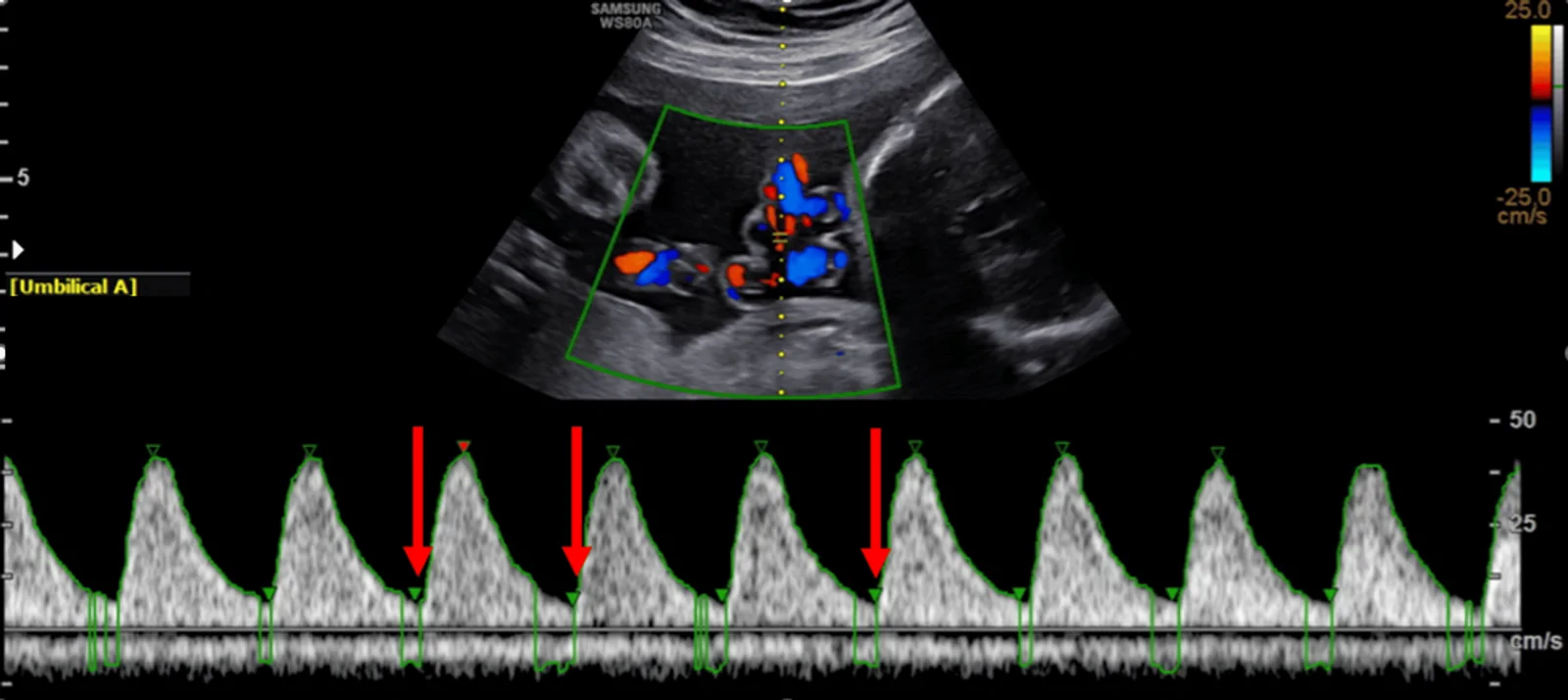
Umbilical artery Doppler ultrasound demonstrating reduced end-diastolic flow. The arrow indicates reduced end-diastolic flow within the umbilical artery waveform. The pulsatility index (PI) of 1.69 is above the 95th centile for 30+3 weeks' gestation, indicating increased placental vascular resistance consistent with placental insufficiency.

An ultrasound scan performed at 29+1 weeks confirmed early-onset severe FGR, with an estimated fetal weight at the 0.2nd centile. Amniotic fluid volume and fetal Doppler studies were normal at that stage. A repeat assessment one week later, at 30+3 weeks, demonstrated abnormal umbilical artery Doppler indices (Figure 1). At the same visit, her blood pressure was elevated at 153/84 mmHg, and significant proteinuria was identified with a urine protein-creatinine ratio (PCR) of 687 mg/mmol (reference range <30 mg/mmol). Computerised cardiotocography (cCTG) did not meet Dawes-Redman criteria owing to the absence of periods of high variation [7]. She was subsequently admitted for maternal blood pressure control and enhanced fetal surveillance.

Oral Labetalol was commenced at 200 mg twice daily and titrated to 200 mg four times daily for blood pressure control. Despite this, she required three additional 200 mg doses of oral labetalol to achieve adequate blood pressure control. Antenatal corticosteroids were administered to promote fetal lung maturity. Due to ongoing concerns regarding maternal and fetal wellbeing, she was transferred to the labour ward approximately 24 hours after admission for one-to-one care and continuous maternal and fetal monitoring.

**Figure 2:**
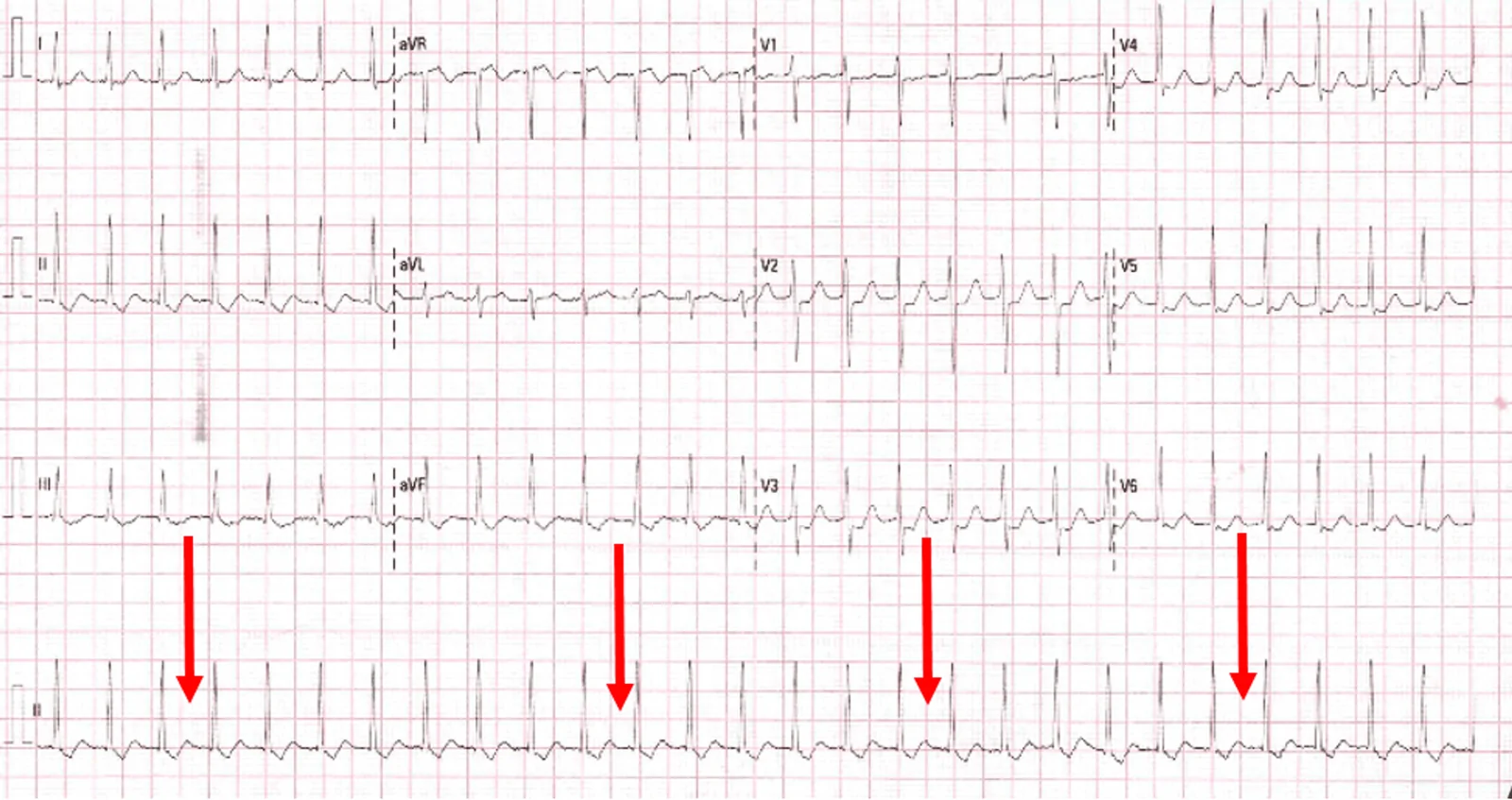
Twelve-lead electrocardiogram demonstrating regular narrow-complex supraventricular tachycardia (SVT) at approximately 164 beats/minute. Arrows indicate the regular narrow QRS complexes with absent discernible P waves, consistent with SVT.

Whilst on the labour ward, she developed sudden-onset tachycardia with a heart rate of 170 beats per minute, associated with breathlessness and chest pain. Her blood pressure was 145/69 mmHg, and oxygen saturations were 99% on room air. An urgent multidisciplinary review involving the obstetric, anaesthetic, and medical teams was undertaken. Vagal manoeuvres were unsuccessful, and 6 mg intravenous adenosine was administered, resulting in successful cardioversion to sinus rhythm. Electrocardiography (ECG) demonstrated a regular narrow-complex tachycardia at 164 beats/minute, consistent with SVT (Figure 2).

Troponin T was mildly elevated at 29 ng/L (reference range <14 ng/L). The initial electrocardiogram demonstrated transient ST-segment changes, which resolved on repeat ECG following restoration of sinus rhythm. N-terminal pro-B-type natriuretic peptide (NT-proBNP) was elevated at 1,700 ng/L (reference range <125 ng/L) and decreased to 700 ng/L after 12 hours. Full blood count, renal function, liver function tests, and electrolytes were within normal reference ranges. Following cardiology review, the troponin elevation was considered most consistent with demand myocardial injury secondary to a combination of sustained tachyarrhythmia in the setting of severe pre-eclampsia. As the patient remained clinically stable and repeat ECGs had normalised, serial troponin measurements were not performed.

Following this episode, continuous fetal monitoring demonstrated an abnormal cardiotocograph (CTG) with reduced variability and recurrent decelerations. Her blood pressure remained elevated, with systolic measurements reaching 180 mmHg. Despite escalation of oral labetalol to 200 mg four times daily, three additional 200 mg oral doses of labetalol, and 20 mg oral nifedipine were required to achieve blood pressure control. Intravenous magnesium sulphate was commenced for seizure prophylaxis as well as fetal neuroprotection [8,9]. Following urgent discussion with the on-call cardiology consultant, obstetric management was prioritised, with plans for a 24-hour Holter monitor and urgent echocardiography following delivery. Given the abnormal antenatal CTG in the context of severe pre-eclampsia and deteriorating fetal status, a decision was made to proceed with lower segment caesarean section (LSCS) after multidisciplinary discussion, including consultation with the neonatology team.

An uncomplicated LSCS was performed under spinal anaesthesia, with an estimated blood loss of 350 mL. Postoperatively, she was transferred to the labour ward high-dependency unit (HDU) for ongoing management of severe pre-eclampsia.

A live female infant was delivered with Apgar scores of 5 at 1 minute and 7 at 5 minutes and required initial resuscitation with inflation and ventilation breaths. The birth weight was 1059 g (0.4th centile), reflecting severe FGR. The infant required admission to the neonatal intensive care unit (ITU) for six weeks, and the clinical course was complicated by respiratory distress syndrome, retinopathy of prematurity, suspected sepsis, and echocardiographic findings of a patent foramen ovale and patent ductus arteriosus. The infant was subsequently discharged home following clinical stabilisation.

**Figure 3:**
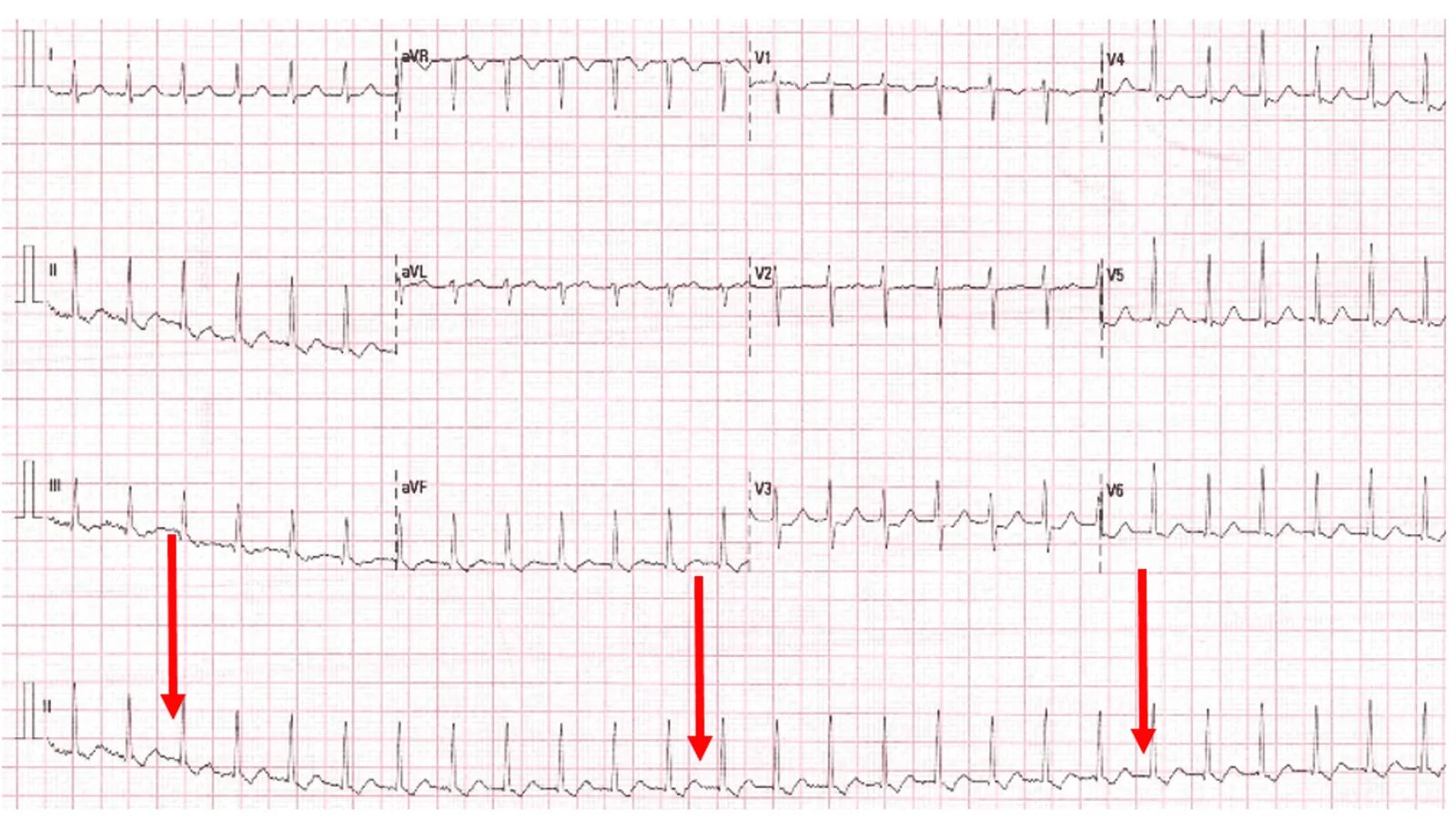
Twelve-lead electrocardiogram obtained 24 hours postpartum demonstrating recurrent regular narrow-complex supraventricular tachycardia (SVT) at approximately 157 beats/minute. Arrows indicate the regular narrow QRS complexes with absent discernible P waves, consistent with SVT.

Twenty-four hours after delivery, while in the postnatal ward, she experienced a further episode of palpitations, breathlessness, and chest pain. Her heart rate was 160 beats per minute and blood pressure was 145/70 mmHg. Electrocardiography demonstrated a narrow-complex tachycardia with absent P waves, consistent with SVT (Figure 3). Vagal manoeuvres were unsuccessful. Administration of 6 mg followed by 12 mg intravenous adenosine terminated the arrhythmia only briefly before recurrence, necessitating a further 18 mg dose, which successfully restored sinus rhythm. At this stage, she was on oral labetalol 200 mg four times daily, and her blood pressure was adequately controlled. Following cardiology review, in view of recurrent SVT despite adenosine therapy, the multidisciplinary team (MDT) considered the addition of a further antiarrhythmic agent for rhythm control. As she was already receiving labetalol with satisfactory blood pressure control, introducing a second β-blocker at that stage was not considered appropriate by the MDT. Verapamil 40 mg three times daily was therefore commenced for ongoing rhythm control, and she was transferred to the ITU for closer monitoring and management.

After 24 hours in ITU, she was stepped down to the cardiology ward and subsequently returned to the postnatal ward. Optimising both blood pressure and heart rate control remained challenging despite ongoing multidisciplinary input from the cardiology, intensive care and obstetric teams. Following the introduction of verapamil, her blood pressure became increasingly labile, requiring two additional 200 mg oral doses of labetalol without satisfactory improvement. In view of the challenges in optimising both blood pressure and rhythm control, her case was discussed in a multidisciplinary meeting involving the cardiology and obstetric teams together with the regional Maternal Medicine Network, a specialist multidisciplinary service providing expert advice for women with complex medical disorders during pregnancy and the postpartum period. It was agreed to discontinue verapamil and commence bisoprolol together with enalapril to optimise both rhythm and blood pressure control in the postpartum period. During the following two days, her blood pressure remained variable but subsequently stabilised without the need for further rescue antihypertensive medication. She experienced one further self-terminating episode of SVT on the fifth postpartum day, after which no additional episodes occurred. Her blood pressure and heart rate subsequently stabilised on bisoprolol and enalapril, allowing discharge home.

Transthoracic echocardiography performed during ITU stay on the second postpartum day demonstrated normal left ventricular size and systolic function, with normal right ventricular structure and function. Mild-to-moderate mitral and tricuspid regurgitation were noted, with no significant structural cardiac abnormalities or pericardial effusion. She remains under cardiology follow-up, with a cardiac MRI planned for further assessment. In view of the traumatic nature of her pregnancy and postnatal course, she has also been referred to the Birth in Mind service for psychological support and debriefing.

## Discussion

The rapid progression from early-onset FGR to severe pre-eclampsia is a well-recognised manifestation of placental dysfunction [5]. Early-onset FGR associated with abnormal placental Doppler findings represents a particularly high-risk phenotype requiring close maternal and fetal surveillance [6,10]. The coexistence of new-onset SVT during a period of significant maternal and fetal deterioration added substantial complexity to management in this case.

Emerging evidence suggests that SVT in pregnancy is associated with an increased risk of adverse maternal outcomes, including in-hospital mortality, cardiogenic shock, requirement for mechanical circulatory support, and hypertensive disorders such as pre-eclampsia and eclampsia [11]. However, there remains limited literature describing recurrent new-onset SVT occurring alongside severe early-onset placental disease and pre-eclampsia, particularly when refractory to initial doses of adenosine and requiring escalation to multiple antiarrhythmic agents. This made clinical decision-making in our case particularly challenging, requiring close multidisciplinary collaboration to optimise both maternal cardiovascular status and obstetric management. Although observational studies have demonstrated an association between SVT and hypertensive disorders of pregnancy [11], the available evidence is insufficient to establish a causal relationship. Whether the coexistence of these conditions represents a true pathophysiological association or a coincidental occurrence remains uncertain, highlighting the need for further research to better understand this relationship and guide management.

The majority of arrhythmias in pregnancy are benign and require minimal intervention [2]. SVT remains the most frequently encountered sustained tachyarrhythmia in pregnancy [2]. Current management guidelines recommend vagal manoeuvres as first-line treatment, followed by intravenous adenosine where required [12]. In cases of hemodynamic instability or failure of pharmacological therapy, electrical cardioversion (ECV) is considered safe and effective throughout pregnancy [12]. Importantly, no significant adverse fetal effects have been reported following ECV, although fetal assessment with cardiotocography is recommended after the procedure [12]. In selected cases, pregnancy itself may act as a precipitating factor for arrhythmia; therefore, delivery may occasionally be a therapeutic intervention [12].

A particularly unusual aspect of this case was the persistence of recurrent SVT episodes in the postpartum period, resulting in ongoing hemodynamic instability and admission to the intensive treatment unit (ITU). While many pregnancy-associated arrhythmias improve following delivery, this patient's clinical course demonstrated that significant cardiac morbidity may persist beyond the immediate obstetric period. Furthermore, the arrhythmia proved relatively resistant to standard therapy. In a study by Elkayam et al., successful termination of paroxysmal SVT was achieved with 6 mg or 12 mg adenosine in approximately 89% of cases [13]. In contrast, our patient required escalation to 18 mg on one occasion before restoration of sinus rhythm, highlighting the recurrent and relatively refractory nature of her arrhythmia. This may reflect the complex interplay between severe pre-eclampsia, fluid shifts, autonomic changes, and myocardial stress occurring in the peripartum period. However, there is currently no direct physiological evidence to establish a causal relationship between severe pre-eclampsia and refractory SVT, and this hypothesis remains speculative. Alternative contributors to postpartum SVT, including rapid haemodynamic changes following caesarean delivery, increased sympathetic activation, autonomic fluctuations, electrolyte disturbances, postoperative physiological stress, and the unmasking of previously unrecognised arrhythmogenic substrate, should also be considered [12]. Further research is required to better define the relationship between hypertensive disorders of pregnancy and SVT.

Finally, this case highlights the critical importance of multidisciplinary management in complex high-risk pregnancies. The successful management of this patient relied not only on timely obstetric intervention but also on ongoing cardiovascular assessment and critical care support. The involvement of specialist maternal medicine and cardio-obstetric teams is increasingly recognised as an important component of managing complex maternal cardiac conditions during pregnancy [4,14]. The case further underscores the need for continued vigilance in the postpartum period, as significant maternal complications may persist or even emerge following delivery despite removal of the pregnancy-related trigger. Careful postnatal monitoring and appropriate specialist follow-up are therefore essential components of comprehensive care in similar cases.

## Conclusions

This case illustrates the complexity of managing concurrent severe placental dysfunction and maternal SVT in pregnancy. The combination of pre-eclampsia, severe FGR with abnormal placental Doppler findings and new-onset maternal arrhythmia required careful assessment of both maternal and fetal status, with ongoing reassessment as the clinical picture evolved.

A key learning point from this case is the importance of early multidisciplinary involvement and timely escalation when maternal cardiac complications occur alongside significant obstetric pathology. Coordinated care involving obstetric, cardiology, anaesthetic, critical care, neonatal and specialist maternal medicine teams was essential in the management of this complex presentation. The challenges encountered in this case highlight the value of early involvement of regional specialist services when managing rare and high-risk maternal conditions, particularly in settings where specialist expertise may be limited.

Importantly, this case demonstrates that delivery is not always the definitive solution to complex obstetric pathology. Despite delivery of the fetus, maternal cardiovascular concerns persisted and required ongoing surveillance, specialist follow-up and careful optimisation of medical therapy. In addition, persistent maternal concerns regarding fetal wellbeing may warrant consideration as a trigger for reassessment, particularly when concerns continue despite initially reassuring clinical evaluations. While the diagnostic value of maternal perception cannot be established from a single case, this case highlights the importance of maintaining clinical vigilance and considering repeat assessment when concerns persist or when there are evolving clinical changes. The prolonged physical and emotional impact of severe maternal and neonatal complications also highlights the importance of appropriate postnatal debriefing and psychological support.

This case reinforces that management of multifaceted obstetric presentations requires an individualised approach, balancing maternal cardiovascular stability, fetal well-being and long-term maternal recovery.
